# Self-template manufacturing of on-skin electrodes with 3D multi-channel structure for standard 3-limb-lead ECG suit

**DOI:** 10.1038/s41378-024-00838-7

**Published:** 2024-12-16

**Authors:** Wentao Wang, Longsheng Lu, Huan Ma, Zehong Li, Xiaoyu Lu, Yingxi Xie

**Affiliations:** 1 School of Automotive and Mechanical Engineering, Changsha University of Science and Technology, Changsha, 410114 China; 2 Guangdong Key Laboratory of Precision Equipment and Manufacturing Technology, South China University of Technology, Guangzhou, 510641 China; 3 Guangdong Cardiovascular Institute, Guangdong Provincial People’s Hospital, Guangzhou, 510080 China

**Keywords:** Electronic properties and materials

## Abstract

Wearable electrocardiogram (ECG) devices are the mainstream technology in the diagnosis of various cardiovascular diseases, in which soft, flexible, permeable electrodes are the key link in human-machine interface to capture bioelectrical signals. Herein, we propose a self-template strategy to fabricate silver-coated fiber/silicone (AgCF-S) electrodes. With a simple dissolving-curing-redissolving process, the polyvinyl acetate shell around the AgCF core is in-situ removed to form a three-dimensional (3D) multi-channel structure. The conductive fibers overlap each other and pass through the silicon substrate in a network state, so that the electrode can be bent to 180° or stretched to 30%. The 3D multi-channels in AgCF-S adhesive is further coupled with a Kirigami-design structure of flexible substrate, to maintain high flexibility without sacrificing air-permeability, enabling an excellent water evaporation rate of 1.8 μg/mm^2^/min, and non-allergenic adhere on pigskin after 24 h. Combined with the self-developed standard 3-limb-lead ECG suit, multi-lead signals with high signal-to-noise ratio (SNR) and low variance (*σ*^2^), can be transmitted in real-time via Bluetooth and displayed in the client. Typical heart diseases such as coronary, arrhythmia, myocardial infarction, etc., are detected by our ECG equipment, revealing a huge promise in future medical electronics.

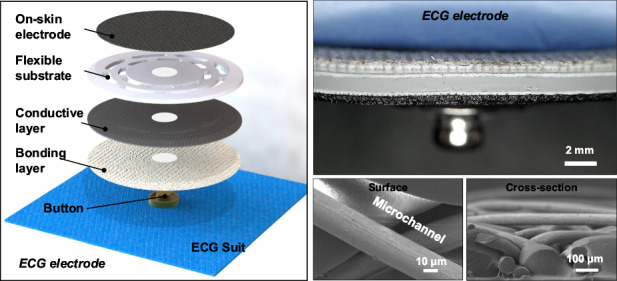

## Introduction

Cardiovascular disease (CVDs) is the leading cause of global mortality, accounting for 31% of all fatalities each year, and unfortunately this situation has been exacerbated by aftermath of the COVID-19 pandemic^1,2^. Early diagnosis of CVDs relies on electrocardiogram (ECG), a valuable noninvasive tool for monitoring cardiac potentials^3,4^. Over the past decade, the appearance of outdated rigid and square-shaped ECG devices has become soft and skin-friendly, with easy-to-detect features that are closer to human body^5^. One of the representative mainstream technologies is wearable ECG devices, which can be attached to non-flat and dynamic skin, and perform 24-hour cardiac monitoring out-of-hospital^6,7^. Up to now, wearable ECG devices are roughly divided into three categories^8^. Traditional devices consisting of rigid silicon integrated circuits (ICs) are packaged into fabrics with an elastic layer for skin contact and signal collection^9^. The fatal drawback of this manner lies in poor wearing comfort and heavy physical burden. Another cutting-edge design uses flexible components, demonstrating a high degree of adaptability but limited lifespan and reliability^10^. As a trade-off, wearable ECG devices with a rigidity-flexible balance of mechanical properties, where the front-end unit is composed of soft patches and the back-end hardware is based on brittle ICs, providing an ideal platform for integration into daily necessities such as clothing, seats, or beds^2^.

Given that direct contact between human-machine interfaces is a key link for the stable capture of physiological signals, an overwhelming challenge for wearable ECG devices is to fabricate on-skin ECG electrodes^11–13^. By far the most popular type is the silver/silver chloride (Ag/AgCl) gel electrode. Over time, yet, the decay of wet gel leads to abnormal signals, i.e. distortion, noise and artifacts, thus lessening the long-term use^14^. The groundbreaking research on textiles such as knitting, weaving and embroidery makes ECG electrodes easy to assemble all-in-one devices by a space-optimized and personalized shape-customizable way^15–17^. Despite a series of good jobs, there are still two big puzzles: i) the bioelectric signals collected by these electrodes are usually of low quality, due to high impedance and poor adhesion at the electrode-skin interface; and ii) the unstable electrode-skin interface may undergo unpredictable changes or even fail, in view of the inevitable deformations encountered in daily life^18–20^.

To address these limitations, different approaches including e-beam or laser processing, thin-film deposition and etching, are widely used to fabricate ECG electrodes^21–23^. Prototype devices such as elastic nanocomposites consist of silver nanowires (AgNWs)^23,24^/nanoparticles (AgNPs)^25^, graphene^26,27^ and carbon nanotubes (CNTs)^10^, or shape-adaptive wavy, cellular and helical geometries^28–31^. Other Kirigami structures, with a topological effect, is also beneficial for enhancing human-computer interaction depend on the shape reconfigurable ability^32–35^. Although most of these strategies work well, further progress is being slowed down by time-consuming and high-cost technical route. At the same time, skin irritation or discomfort may still occur during long-term cardiac monitoring^36^. Therefore, endowing on-skin ECG electrodes with good breathability to reduce side-effects, while keeping flexibility and self-adhesion to cope with external deformation, remains a daunting challenge.

The self-template method is commonly used in traditional chemical reactions to prepare hollow nanoparticles, which involves the synthesis of micro-nano templates first, followed by the removal of intermediate structures. In this work, soft, flexible, and permeable silver-coated fiber/silicone (AgCF-S) electrodes are fabricated using a self-template method, including dissolving-curable redissolution steps. Differently, the polyvinyl acetate (PVA) is wrapped on AgCFs, and then water-dissolved in silicone elastomer. The template is AgCFs, which not only serves as a skeleton, but also directly participates in the formation of 3D muti-channel structures, thus endowing the dry adhesive with high permeability and low impedance. By kirigami-shape design of soft substrate and assemble in a layer-to-layer manner, on-skin ECG electrodes are further integrated into standard 3-limb-lead ECG suit. The robust function is verified by capturing the I–III, aVR, aVL, aVF, and V4 lead data with high signal-to-noise ratios (SNRs) in typical scenarios such as motion tracking, sleep monitoring and rehabilitation training. Combined with remote transmission and visualization technologies, three diseases including arrhythmia, coronary heart disease and myocardial infarction are detected, reflecting the great potential of standard 3-limb-lead ECG suit in first-line medical electronics.

## Experimental section

### Material and processing parameters

The on-skin adhesives with 3D multichannel structure were prepared by self-template method. Specifically, the silver-coated fabrics (AgCFs, Zhiyuan Xiangyu Textile Co., Ltd., China, thickness: 0.2 mm) were cut into 40-mm diameter circles, and then cleaned in a plasma cleaning machine (PT-2s, Sanhe Boda Electromechanical Technology Co., Ltd., China) with oxygen atmosphere for 120 s. The resin powder of polyvinyl alcohol (PVA, 1788, China National Pharmaceutical Group) was stirred in deionized water at 90 °C until completely dissolved, with solution concentrations of 2.5 wt%, 5 wt%, 7.5 wt%, and 10 wt%, respectively. Subsequently, about 0.5 ml of PVA solution was uniformly coated on AgCFs, with an average density of 0.398 L/m^2^. These samples were subsequently put in a heating oven (DHG-9070A, Yiheng scientific instrument, China) until the PVA solution was dried into a film. Silicone rubber (Ecofelx-0030, SmoothOn, USA) and medical silicone gel (XY-8060, Unison Technology Co., Ltd., China) were mixed according to the mass ratio of 3:7, 2:8 and 1:9, respectively. The mixed silicone was evenly applied onto AgCFs for defoaming, and a scraper was used to remove excess silicone. The above operations should be completed within 10 minutes at 25 °C, and heated for 15 mins in a drying oven at 35 °C. After solidification, the on-skin adhesive was placed in hot water to dissolve the PVA coating, and completely dried at room temperature for 2 days. For convenience, the sample code for the AgCF after PVA self-template dissolution and mixed silicone pouring is called AgCF-S.

The flexible substrate is fabricated according to Kirigami-design patterns, including blank, serpentine groove, circular groove, and diamond groove with 1, 2 and 3mm-slot width. All the substrates are fabricated using the reverse molding method. Specifically, the plastic molds (Polylactic acid material, diameter: 50 mm, thickness: 5 mm) were pre-manufactured by a UV cured 3D printer (SLA2100T, Zhongrui Technology Co., Ltd., China), the internal region (diameter: 40 mm, thickness: 2 mm) were designed according to Kirigami patterns. Silicone rubber (Ecofelx-0030, SmoothOn, USA) was poured into mold by mixing A and B colloidal components (1:1 weight ratio). After 10 mins of vacuuming, the mixture was completely spread out and then heated at 80 °C for 4 hours. The Ecoflex is stripped from mold after curing, thus the flexible substrates were formed with specific Kirigami structures.

### Characterization

Surface and cross-section morphology were observed by a field emission scanning electron microscopy (FE-SEM, Carl Zeiss) with a working distance of 15 mm and a 5 KeV electron beam. Elemental spectra were recorded in 0.1 eV step sizes with a pass energy of 26 eV. Sheet resistance was measured by a four-point conductive meter (RC3175, EDTM), each sample was tested with five different positions. The self-built dynamic frequency impedance device was powered by a digital source meter (CC2450; Keithley, USA). The vibration was recorded by a triaxial force sensor (GSV-4; ME-Messsysteme, Germany). Impedance measurement was done by inductance resistance and capacitance (LCR) digital bridge (TH2838; Tonghui, China). Adhesion was tested on a tensile testing machine (ZQ-990B; Zhizhi Company, China) with data recorded by a high-precision force sensor (GSV-4; ME-Messsysteme). The AgCF-S dry adhesive was cut to the appropriate size with an adhesion area of 1×1 cm^2^, and a 10 kPa pressure was applied during adhesion. During the test, tangential displacement was applied at a speed of 5 mm/min to test the tangential adhesion force, and the sample was peeled off at 90° from the Si-wafer, Si-rubber, and pigskin at a normal speed of 5 mm/min. The water evaporation rates under pure AgCF, AgCF-S and 3 M gel electrode were tested to reflect the air permeability. Three electrodes were cut into same size to cover the bottle filled with water, and then measure the overall mass using a precision balance (106DUH/AC, Mettler Tolledo, Switzerland). All the samples were placed in a drying oven at 36 °C to test the mass every 30 mins in 3 hours. The electrodes are pretested in professional electrocardiogram machine (CARDIOVIT CS-200 Excellence ErgoSpiro, SCHILLER) to demonstrate applicability.

### Hardware design of ECG system

A low-power 3-channel digital electrocardiogram chip (ADS1293, Texas Instruments, USA) was integrated with a Wilson potential generation network and a driver amplification circuit. The main control (STM8L151, STF Semiconductor Company) coped with Bluetooth (CC2541, Texas Instruments) were employed as the communication part. A Memory (NOR Flash, Huabang Electronics) was selected for motion detection and data storage. The hardware based on the operating system abstraction layer (OSAL, Texas Instruments, USA) was controlled by a microcontroller (Arduino, Italy). The master program was set up to read data, and send to a visualization software program that is based on the virtual instrument LabVIEW. The analog part was powered by a low voltage differential linear regulator (LDO), while the digital part was powered by a switching mode of DC voltage conversion circuit. The analog and digital voltages were higher than 2.7 V and 1.8 V, respectively. The ECG front-end was set to a sampling rate of 320 sps, and the main control was set to receive data every 2 interrupts, with a sampling rate of 160 sps. The format of ECG data was hexadecimal, with each 3-byte group, corresponding to lead data of I, II, and III. These data were sent in binary format and undergone AD conversion by the central processing unit (CPU, Siemens, Germany), and then wirelessly transmitted by Bluetooth and displayed by computer or telephone client.

## Results and discussion

### Overall design of standard 3-limb-lead ECG suit

**Fig. 1:**
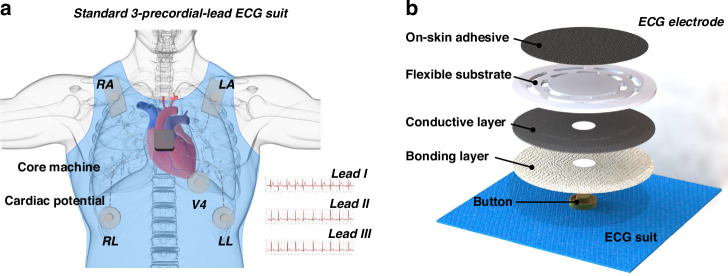
Overall design of the standard 3-limb-lead ECG suit. **a** Schematic diagram of the standard 3-limb-lead ECG suit consisting of a core machine and ECG electrodes. **b** Layer-by-layer diagram of ECG electrode

The ECG monitoring is an important method to analyze cardiac diseases such as irregular heart rate and myocardial ischemia^37^. As shown in Fig. 1a, a total of five electrodes are arranged in the corresponding positions of a standard 3-limb-lead ECG suit to capture the cardiac signals. On-skin adhesives are set in medically prescribed positions, then rely on a core machine for data collection, synchronization, and transmission. Among them, leads I and II represent the potential difference between the left arm (LA) and right arm (RA), as well as the left leg (LL) and right leg (RL), respectively. The potential difference between the LL and LA belongs to lead III. Other three leads, aVR, aVL and aVF, are obtained via the arithmetic gauge:

1 $${aVR}=-\frac{I+{II}}{2}$$

2 $${aVL}=\frac{I-{II}}{2}$$

3 $${aVF}=\frac{{II}-I}{2}$$

The schematic diagram of layer-by-layer structure in ECG electrode is displayed in Fig. 1b. The ECG electrode is divided into five parts, including on-skin adhesive, flexible substrate, conductive layer, bonding layer, and button. The back of flexible substrate is opened and the buttons is acted as a medium to contact each layer, then connected to ECG suit to transmit bioelectrical signals. On-skin adhesive is made of self-template method and flexible substrate is designed with Kirigami structure through a casting-and-peeling process. A total of seven different Kirigami structures are made, in favor of the conformal contact at human-machine interface. The detailed structural analysis will be discussed in the following sections. Based on self-developed hardware and software, the standard 3-limb-lead ECG suit can maintain low impedance and high air permeability while being suitable for functional verification in multiple dynamic/static scenarios.

### Self-template manufacturing of on-skin adhesives

**Fig. 2:**
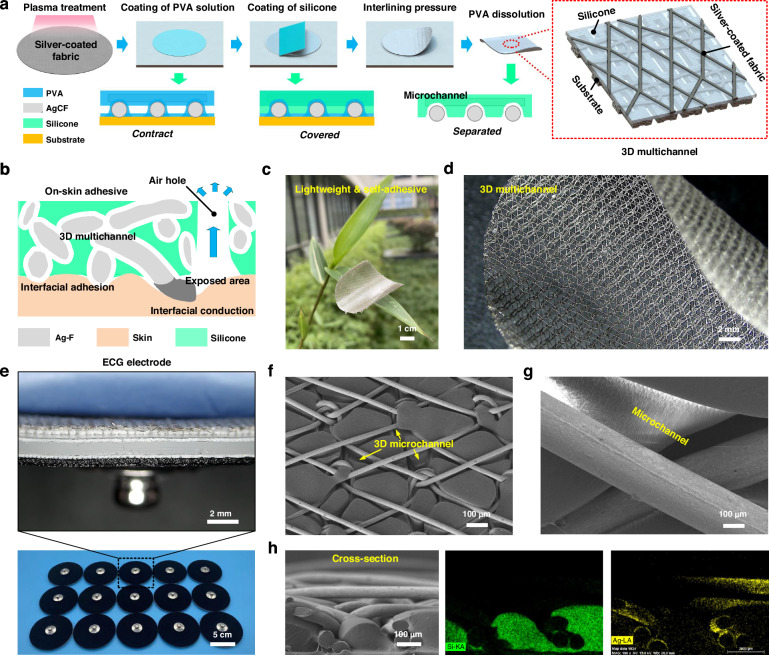
Self-template manufacturing of on-skin electrodes with 3D multi-channel structure. **a** Schematic diagram of the self-template manufacturing process (left), and the on-skin adhesives have 3D multi-channel structures (right). **b** Schematic diagram of interfacial behaviors between electrode-skin (up) and the images of ECG electrodes (down). **c** An on-skin adhesive is placed on a leaf and (**d**) the enlarged optical image. **e** Optical image of ECG electrodes. **f**, **g** The surface morphology of on-skin adhesive. **h** Cross-section view of on-skin adhesive and the element mapping results

An ECG electrode capable of direct medical use is composed of multifunctional layers, the most critical of which is the top layer in direct contact with human skin. As shown in Fig. 2a, the on-skin adhesive with 3D multichannel structure was prepared by self-template method, which consists of three steps: i) PVA solution was coated on pure AgCF and dried into a sheet; ii) silicone mixture is poured on the AgCF sheet; and iii) the heat-cured sheet is placed in water to dissolve the PVA coating. Fig. S1 demonstrates the network structure of AgCF is composed of weft and warp lines. The weft is a single fiber with a diameter of ~35 μm. The warp is made up of four fibers, and the connecting points form the electrical paths between the layers. The interfacial behaviors between the ECG electrode and skin are depicted in Fig. 2b. In a previous study, we investigated a mixed silicone of medical gels and Ecoflex to confer excellent self-adhesion on ECG electrodes, in which the medical gel provides interface adhesion, and Ecoflex improves the overall strength. Again, this silicone formula is used in the encapsulation of the electrodes, except that our focus this time is on the self-template manufacture of 3D multi-channel structure. These multi-channels at the human-machine interface are evenly distributed within a 3D network, resulting from the dissolution of PVA “shell” during the self-template process. The exposed fibers serve as electrical connections, and the internal fibers are beneficial for the continuity of adjacent silicone “islands”, so that the interface contact area is increased while reducing the risk of detachment. In Fig. 2c, an on-skin adhesive is placed on a leaf to demonstrate its light-weight and self-adhesion properties. The internal AgCFs are interwoven, thus can be bent to 180° or stretched to 30%, making it sufficient to cope with deformation encountered during daily use (Fig. 2d). As shown in Fig. 2e, all the layers are tightly bonded to each other. Due to the low-cost self-template process, as well as the simple assembly process, roll-to-roll manufacturing can be adopted to achieve large-scale production.

The surface morphology of AgCF-S adhesive is illustrated in Fig. 2f-g. The longitude and latitude intersection are a vertical water vapor channel, so that the ECG electrode is air permeable no the need for additional drilling. In contrast, on-skin adhesives without self-template fabrication are relatively flat (Fig. S2). Due to the continuous distribution of silicone layer, there is no exposed AgCFs and multi-channel formation, leading to the poor contact impedance in ECG electrodes. Figures 2h and S3 show the cross-section structure of on-skin adhesive with a thickness of ~600 μm, and the diameter of internal 3D multichannel increases as the concentration of PVA solution increases, so the water vapor permeability is highly improved. As the concentration of PVA solution is 7.5%, the 3D multichannel pass directly through the silicone layer, endowing the electrode with great water vapor permeability. The cross-sectional element mapping shows that the microchannel penetrates completely from top to bottom, and has good consistency in shape, structure, and size. Therefore, in some scenarios where on-skin adhesives are worn for long-term monitoring, the negative effects such as skin allergies, redness, and swelling can be effectively avoided.

**Fig. 3:**
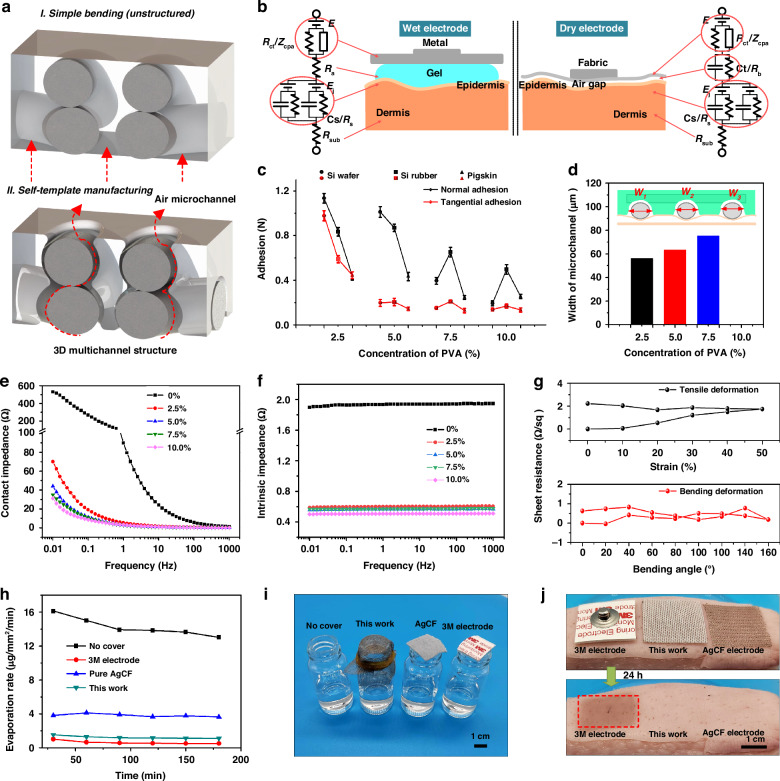
Electrical and mechanical properties of on-skin adhesives. **a** Schematic diagram of air-escaping path in 3D multi-channel structure. **b** Schematic diagram of interfacial impedance between electrode and skin. **c** Normal and tangential adhesion of AgCF-S electrode against to Si-wafer, Si-rubber, and pigskin. **d** Channel width, **e** Intrinsic impedance and **f** Contact impedance of on-skin adhesive varies with increasing content of PVA solution. **g** Sheet resistance of AgCF-S electrode at stretching and bending deformations. **h**, **i** Commercial 3 M electrode, pure AgCF, and AgCF-S electrodes are covered on bottles containing water to estimate water evaporation rate and (**j**) attached to pigskin for 24 h

According to the above structural analysis of on-skin adhesives, the principle of air-escaping path is depicted in Fig. 3a. In contrast to traditional physical blending, 3D multi-channels inside of silicone elastomer are distributed along the AgCFs, allowing for easy excretion of human sweat. Due to the capillary force of the fiber, sweat is easily absorbed by the AgCF-S adhesives and thus forcefully discharged (Fig. S4). The interfacial adhesion model is demonstrated in Fig. 3b. The electrical network of skin is divided into dermis and epidermis. The dermis, partly due to cells, exhibits good electrical conductivity and can be equivalent to *R*_sub_. The epidermal part is mainly the stratum corneum, so that can be equivalent to a capacitor composed of resistance *R*_s_ and capacitance *C*_s_ connect in parallel. According to the contact mode, the on-kin electrodes are divided into dry and wet types. To obtain a high-quality ECG signal, two conditions need to be met: i) a low electrode-skin contact impedance to reduce interference and ii) a low intrinsic impedance to reduce signal attenuation. The impedance *Z*_a_ and *Z*_b_ of the captured signal can be expressed as follows:

4 $${Z}_{a}=\left({R}_{{ct}}{\rm{||}}{Z}_{{cpa}}\right)+{R}_{a}+\sum\left[{R}_{s}{\rm{||}}\left(-j\frac{1}{\omega {C}_{s}}\right)\right]+{R}_{{sub}}$$

5 $${Z}_{b}=\left({R}_{{ct}}{{||}}{Z}_{{cpa}}\right)+\left[{R}_{b}\parallel \left(-j\frac{1}{\omega{C}_{t}}\right)\right]+\sum \left[{R}_{s}{\rm{||}}\left(-j\frac{1}{\omega {C}_{s}}\right)\right]+{R}_{{sub}}$$

where *Z*_a_ and *Z*_b_ are the interface impedances of wet electrode and dry electrode against skin respectively. The *||* is the parallel calculation, −*j* is the imaginary part of impedance, and *ω* is the angular frequency. According to the above analysis, the contact system of dry electrode is more complex compared with the wet electrode. In the past, Holm et al. proposed that for a conductor with a circular cross-section, the resistance *R*_b_ can be expressed as a function of the contact radius *a* and the conductor resistivity *ρ*:

6 $${R}_{b}^{{\prime} }=\frac{\rho }{2a}$$

The 3D multi-channels in AgCF-S adhesives deform together with and skin, to produce a conformal area *S*. There is an ultra-thin gap at the conformal region of electrode-skin interface, forming a gap resistance and capacitance. Thus, the corresponding part of formula (5) can be changed as follows:

7 $$\left[{R}_{b}\parallel \left(-j\frac{1}{\omega {C}_{t}}\right)\right]=\frac{{\rho }_{1}{t}_{1}}{S}+\left[\frac{{\rho }_{2}{t}_{2}}{S}\parallel \left(-j\frac{{t}_{2}}{\omega \varepsilon S}\right)\right]$$

where *ρ*_1_ and *t*_1_ is the resistivity and thickness of conductive layer, the *ρ*_2_, *t*_2_ and *ε* are the resistivity, thickness, and dielectric constant of interfacial gap, respectively. Since the AgCFs are interconnected, the contact area *S* can be written as:

8 $$S=2b\sqrt{\frac{4{F}_{1}}{\pi b}\frac{\frac{1-{\mu }_{1}^{2}}{{E}_{1}}+\frac{1-{\mu }_{2}^{2}}{{E}_{2}}}{\frac{1}{{r}_{1}}-\frac{1}{{r}_{2}}}}$$

where *b* is the equivalent contact length in each area, *F*_1_ is the normal force in the region. The *μ*_1_ and *μ*_2_ are Poisson’s ratio of AgCFs and skin, respectively. The *E*_1_ and *E*_2_ are elastic modulus of AgCFs and skin, respectively. The *r*_1_ and *r*_2_ are the curvature radium of AgCFs and skin, respectively.

The normal adhesion force of on-skin adhesive is given according to force relationship:

9 $${F}_{n}={F}_{{nw}}+{F}_{1}+{F}_{2}\le {F}_{{nmax}}$$

where *F*_n_ is the normal adhesive force in the contact region, which varies within a certain range, and not greater than the maximum normal adhesive force *F*_nmax_. The *F*_nw_ is the normal tearing force in the contact region. The *F*_2_ is the reverse force generated by the viscous layer of silicone elastomer. The *F*_1_ and *F*_2_ are related to the compressive degree of interface force. It can be seen from the formula that the *F*_1_ changed into the largest value when the *F*_*nw*_ is 0, and the external tear is unfavorable to the contact interface.

Normal adhesion is provided by the van der Waals interaction between the electrode-skin interface, while capillary adhesion and self-adhesion effects are both existed in 3D multi-channel structures, given by:

10 $${F}_{{nmax}}={{S}_{1}\sigma }_{v}+{\sum {S}_{2i}\sigma }_{{ci}}$$

where *S*_1_ and *σ*_v_ are the adhesion area and normal adhesion strength under van der Waals action, *S*_2i_ and *σ*_ci_ are the differential adhesion area and differential adhesion strength under capillary force. When the interfacial contact region is a circle with radius *R*_i_, there is

11 $${{S}_{2i}\sigma }_{{ci}}=\pi {R}_{i}^{2}\gamma \frac{\cos {\theta }_{1}+\cos {\theta }_{2}}{{t}_{3i}}$$

where the *γ* is the surface tension. The *θ*_*1*_ and *θ*_*2*_ are contact angles of electrode and skin, respectively. The *t*_3i_ is thickness for differential contact area. In the 3D multi-channel structures, the interface adhesion mainly depends on the van der Waals action and capillary force, so the following conclusions can be drawn: i) normal adhesion increases with the increase of van der Waals effect; ii) the contact impedance decreases with the increase of the interface area; iii) the sweat between the skin-electrode interface helps to reduce the contact impedance.

According to the above analysis, the adhesion is very important for the contact impedance. Figure 3c depicts the normal and tangential adhesion of AgCF-S electrode against to skin. We cut the electrode sheet with the size of 2 × 2 cm^2^ and applied 2.5 kPa pressure to adhere onto the pork skin. Normal and tangential stretching is then carried out at a speed of 5 mm/min. The normal and tangential adhesion strength are both decreased as the concentration of PVA solution increased. When the concentration of PVA solution is 2.5%, the on-skin adhesive shows the highest value because the silicone layer is almost continuous. The increase of PVA content is benefit for the formation of internal 3D multichannel. In other words, the increase in air permeability will inevitably lead to a partial sacrifice of adhesion properties. We measured the surface topography under different PVA content (Fig. S2), and summarized the channel width, as shown in Fig. 3d. With the increase concentration of PVA solution, the channel width increased, and a maximum of 73.8 μm is obtained at 7.5% content. However, the microchannel is too large to form a regular shape at 10% content. As shown in Fig. 3e, the intrinsic impedance of on-skin adhesives is significantly reduced in the frequency range of 0.01-100 Hz. A similar situation occurs in the contact impedance (Fig. 3f). Due to the saturation of conductive region between the electrode-skin interface, the contact impedance is essentially at the same level as the increase concentration of PVA solution. Furthermore, a device was built to test the dynamic stability of contact impedance, while the AgCF-S and 3 M electrodes exhibit strong interfacial stability (Fig. S5).

To verify the resistance stability of AgCF-S electrode when subjected to external changes, the cyclic curve of resistance change rate is tested by stretching and bending experiments, as shown in Fig. 3g. When stretched from 0% to 50% elongation, the resistance changes slightly. In the recovery stage, due to the hysteresis of structural reconstruction, the internal resistance of conductive network is further increased, but it is still at a low level. When the electrode is bent, the resistance changes weakly and has little effect on its resistance. In Fig. 3h-i, we attached samples on the bottles containing pure water to study the water evaporation at room temperature. Due to the encapsulation of self-adhesive silicone elastomer, the breathability of AgCF-S electrode (1.8 μg/mm^2^/min) is smaller compared to that of pure AgCF (3.9 μg/mm^2^/min) is relatively small, but it is still 1.25 times larger than 3 M electrode (0.8 μg/mm^2^/min). In other words, to obtain the adhesion ability of the electrode, a certain amount of air permeability will be sacrificed. Interestingly, the AgCF-S electrode achieves a good balance between permeability and adhesion. Using pigskin as a base, we explore long-term wearability and skin sensitization of different samples (Fig. 3j). Compared with the 3 M gel electrode and the pure AgCF electrode, the AgCF-S electrode left the lightest mark on the pigskin after 24 h of bonding. The long-term stability of the ECG electrode including temperature, repeated bending situation, and laundry are investigated in Fig. S6. The ECG electrodes show very little changes of sheet resistance after high-temperature treatment of 80 °C for 5 h, bending for 1000 times (180°) and repeatedly cleaned and dried for 100 times, and the ECG signal remain clearly when reused. Due to the 3D multi-channel structure, the on-skin adhesive exhibits both high permeability and low impedance. Also, it can ensure a certain limit of contact force when the ECG suit is not well fitted to the skin, presenting the huge potential for long-term wear.

### Kirigami design of flexible substrates

**Fig. 4:**
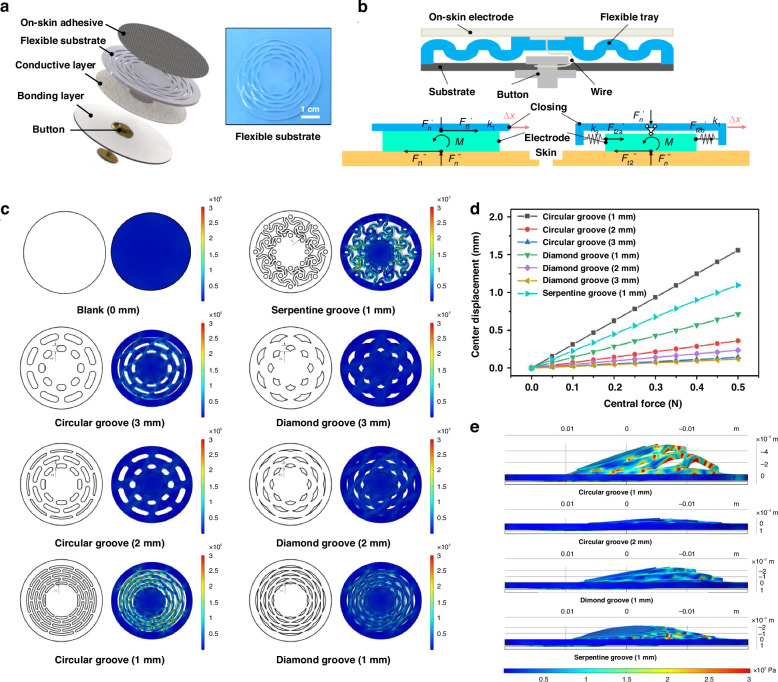
Kirigami design of on-skin ECG electrodes. **a** Schematic diagram of Kirigami-design in flexible substrate. **b** Schematic diagram of force analysis at electrode-skin interface. **c** FEM of mechanical deformation in Kirigami structure of flexible substrate. **d** The central displacements of Kirigami structures under different applied forces. **e** FEM of cross-section deformation in Kirigami structures

Given that some extreme deformations such as stretching, bending, and twisting would inevitably occurs when the AgCF-S electrode is worn on human body. Conformal contact of human-machine interface is the key link to capture the bio-electricity signals. Therefore, a flexible Kirigami-design substrate manufactured by the reverse mold method is employed to ECG electrodes. The detailed process is described in the experiment section. Plastic molds were prefabricated by UV-curable 3D printing. Then, silicone rubber is poured into the mold and a curing-and-demolding process is performed to prepare the flexible substrates with Kirigami-design structures. This method has high accuracy and can achieve a structural scale of 1 mm. In Fig. 4a, the upper part of flexible substrate is attached to an on-skin adhesive and the lower part is connected to a button. Such a layer-by-layer design has three merits: i) cushion the stress through self-adaptive behavior; ii) transfer stress to the suit by the elastic design of metal button; and iii) delivers stable ECG signals to the core machine. The structure diagram of metal button is shown in Fig. 4b. There is only tangential force *F*_*t1*_ between the ECG suit and electrode without the Kirigami structure, which can be used as a spring and represented by tangential force *F*_*t2*_. When the external force *F*_*t1*_ and *F*_*t2*_ are consistent, the mechanical equilibrium conditions of ECG electrode is same as that of human skin. Since the deformation mainly comes from the skin, the driving condition is displacement rather than force. When the external displacement *Δx* is constant, the mechanical relationship can be expressed as:

1) Without a flexible Kirigami-design substrate, the stiffness of clothing *k*_*1*_ is used as the ECG monitoring system and the tangential force *F*_*t1*_ of the electrode-skin interface is given by:

12 $${F}_{t1}=\triangle x{k}_{1}$$

2) With a flexible Kirigami-design substrate, the stiffness of clothing *k*_*1*_, the stiffness of flexible substrate *k*_*3*_, and the stiffness of cloth-electrode system *k*_*ce*_ are regarded as spring systems in series:

13 $${k}_{{ce}}=\frac{{k}_{1}{k}_{3}}{{k}_{1}+{k}_{3}}$$

Then, the tangential force *F*_*t2*_ at the electrode-skin interface is given by:

14 $${F}_{t2}=\triangle x{k}_{{ce}}$$

From the above equation, we can see that the tangential force is relatively small, that is, *F*_t2_ < *F*_t1_. Also, with the decrease of structural stiffness *k*_3_, the tangential force of electrode-skin interface decreases, and the Kirigami structure becomes more stable and difficult to decouple.

The Kirigami-design substrate is good at slowing down the external impact and making up for the displacement. To distinguish the self-adaptive ability of different structures, we set the Kirigami-design structure of flexible substrate as two kinds, namely circular groove, and diamond groove. The design region is a ring area with an outer diameter of 34 mm and an inner diameter of 16 mm, which is successively divided into three kinds of arc-wall with 1-, 2- and 3-mm widths. As a contrast, we also set up a flat and a serpentine structure. All the structural model was simplified and input into COMSOL software for finite element modelling (FEM). The material is silica elastomer, and the mechanical parameters in the database were used as input. The structural grid is tetrahedral, with 104079, 31616, and 18593 grids based on the arc-wall sizes of 1, 2, and 3 mm, respectively.

As shown in Fig. 4c, the Kirigami structure with 1mm-slot width display lower stiffness and larger shape variable than that with 3mm-slot width. Under different applied forces, the central displacements of Kirigami structures are displayed in Fig. 4d. As the lateral force applied to the electrode increases, the displacement is gradually enhanced. Since the high symmetry and small arc arm of Kirigami design, the constraint degree of 1mm-slot width structure is lower compared to 2mm-slot width structure. The flexible substrate with 2mm-slot width shrinks horizontally with minimum turnover. The serpentine structure with 1mm-slot width belongs to a centrosymmetric rather than axisymmetric mode. This structure is unlikely to flip, but it can easily rotate along the axis, leading to adverse effects on interfacial adhesion (Fig. 4e). In Fig S7, the Kirigami-design flexible substrate has good stability, and the strain remains basically stable after 100 times of stretch-recovery cycle at 0-50%. Therefore, we used a circular groove with 1mm-slot width as the Kirigami structure of flexible substrate, which can withstand the shape changes due to its highest variable height. The output waveforms of the AgCF-S and 3 M electrodes are directly compared on the medical-grade ECG machine (Fig. S8). The captured signal by AgCF-S electrodes show obvious PQRST peak, in close to that of 3 M electrode. Furthermore, to test the signal acquisition of AgCF-S electrode after contamination, we immersed it in artificial sweat. After soaking for 5 days, the electrode is not damaged, and only the metal buckle corroded (Fig. S9).

### Integration of standard 3-limb-lead ECG suit

**Fig. 5:**
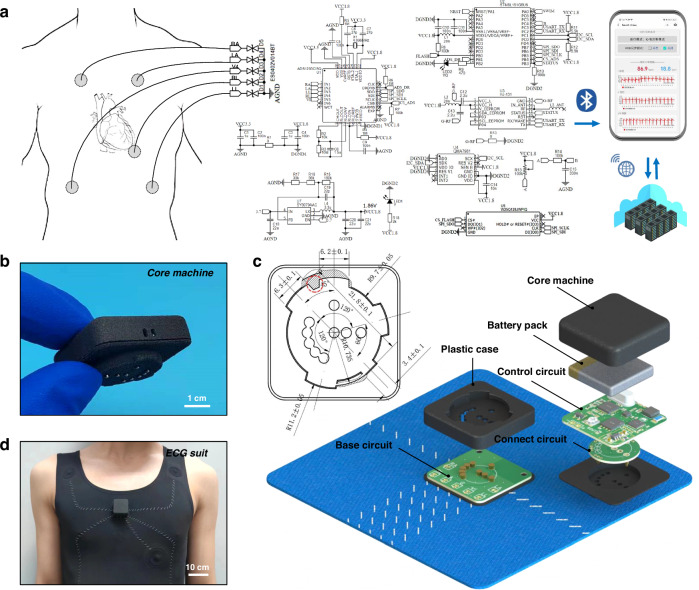
Hardware/software integration design of the standard 3-limb-lead ECG suit. **a** Hardware design of core machine, the ECG signals are displayed by the WeChat program and uploaded to cloud server for storage. **b** Image and **c** schematic layout of core machine. **d** Core machine is fitted to a suit and worn on a volunteer

The ECG electrodes, including on-skin adhesive and flexible substrate, are integrated into the standard 3-precordial lead ECG suit along with the core machine. As shown in Fig. 5a, the hardware part of core machine uses an operational amplifier, as well as a microcontroller for logic control and human-computer interaction. The ECG sensing system includes I, II, III, aVR, aVL and aVF leads, where the differential operation is achieved through amplification of an amplifier, with a built-in large capacity memory for data recording. The chest lead V4 generates the Wilson potential by connecting leads I, II and III to a single point. The ECG signal output by the operational amplifier is converted into a digital signal via a high-speed analog-to-digital converter (ADC). These data are sampled by digital filters to meet different formats, and then stored in integrated operational amplifiers. The hardware design around front-end chips is the technical core of the standard 3-limb-lead ECG suit.

Considering the low-power communication requirements, the digital part uses the DC-DC circuit with 1.8 V power source, and the analog part uses the LDO circuit with 3.3 V power source. The preceding stages of both digital and analog power supplies are decoupled using capacitors. The input of the ECG front-end chip is grounded in parallel with an electrostatic protection diode. Considering the contact impedance of skin-electrode interface and common-mode interference of system, the output amplifier is set, and the suppression signal *V*_RLD_ is expressed as:

13 $${V}_{{RLD}}=-\frac{\left|Z\right|{V}_{{CMOUT}}-(\left|Z\right|+R){V}_{{RLDREF}}}{R}$$

where *|Z|* is the mode of resistance *R*_3_ and *C*_8_ in parallel; *V*_CMOUT_ is common-mode signal output by the common-mode detector; *V*_RLDREF_ is reference voltage in chip. Serial peripheral interface (SPI) is adopted for communication between chip and main control. As a peripheral control and transfer station, the main control communicates with digital interface. A Bluetooth module is powered, and communicated with main control using universal asynchronous transceiver transmitter (UART). The power input of microcontroller adopts PI filter to eliminate ripple, and the working clock is provided by high-frequency RC oscillation circuit. The main power supply is a 120 mAh-3.7 V soft lithium battery with a constant voltage output of 3.3 V. Due to the susceptibility of electrocardiogram signals to electromagnetic interference, ferrite inductors with magnetic seals are used to reduce magnetic leakage. At the same time, a resistor short circuit is used between digital and analog power sources to reduce interference. The hardware is integrated on a printed circuit board (PCB), including the control, communication, storage, processing, power supply and other functions. The visualization software is implemented on the WeChat program to display the ECG data. The ECG data is packaged and classified into different data sequences, and the real value is transmitted back to the user interface through the wireless network. The data storage, signal filtering and time-frequency domain analysis are carried out on the back-end of system, while the analysis, calculation and database building are done by the cloud server.

As shown in Fig. 5b, to facilitate device charging and replacement, the hardware circuit is all integrated in a core machine less than 27.5 × 27.5 × 12mm^3^. The schematic layout of core machine in standard 3-limb-lead ECG suit is depicted in Fig. 5c. This all-in-one device is divided into top and bottom base. The top base is composed of plastic case, battery pack and control circuit, while linked with bottom base with the connect circuit. A 4-tenon rotating tenon structure is adopted between the core machine and ECG suit. The core machine is pressed into the bayonet after 45° clockwise rotation, then the system is powered on. When the ECG suit is not in use or cleaned, the core machine can be easily removed. The noise of the ECG system is less than 30 μV, and the baseline drift is less than 25 μV at 60 s (Fig. S10a-c). Through the steps of power-on, Bluetooth connection, ECG collection and stop, the corresponding current are 0.45, 1.19, 2.66 and 0.77 mA, respectively (Fig. S10b-d). The relevant indicators of the ECG equipment are listed in Table S1. The low current consumption makes the system long endurance and competent for long-term ECG monitoring. In Fig. 5d, a core machine is fitted to the ECG suit and then worn on a volunteer to test the signals. As shown in Fig. S11, the total mass of ECG suit and core machine is about 160 g, thus in favor of reducing the obstacles to human activities. In addition to AgCF-S electrodes, it can also be loaded with 3 M electrodes, reflecting the versatility of our standard 3-limb-lead ECG suit.

### Application of standard 3-limb-lead ECG suit

In response to the demand for multi-lead ECG monitoring, the standard 3-limb-lead ECG suit is operated in various scenarios, to verify the signal-acquisition ability in rehabilitation, telemedicine, dynamic/static monitoring, and disease screening (Video S1). Due to the ECG signals and motion artifacts at limb leads, the useful signal intensity is artificially high. Here, the lead V4 are evaluated with signal-to-noise ratio (SNR) and signal variance (*σ*^2^). Based on the spectral range of ECG signals and the auxiliary fluctuations of front-end chip, a high pass filter, in a 6-order forward-backward manner, is used to obtain useful and noisy signals, respectively. The SNR is given by:

14 $${SNR}\left({dB}\right)=20{\log }_{10}\frac{{{RMS}}_{{useful}}}{{{RMS}}_{{noisy}}}$$

where *RMS*_useful_ and *RMS*_noisy_ is the root mean square of the useful and noise part, respectively. The RMS is the square root of signal values *V*_i_, and the formula can be expressed as:

15 $${RMS}=\sqrt{\frac{\mathop{\sum}\nolimits_{i=1}^{N}{V}_{i}^{2}}{N}}$$

where *N* is the total number of sampling points. Signal variance *σ*^*2*^ is used to describes the variation in the signal waveform, which is given by:

16 $${\sigma }^{2}=\frac{\mathop{\sum}\nolimits_{i=1}^{N}{({V}_{i}-V)}^{2}}{N}$$

where *V* is the mean of all sampling points *V*_i_.

**Fig. 6:**
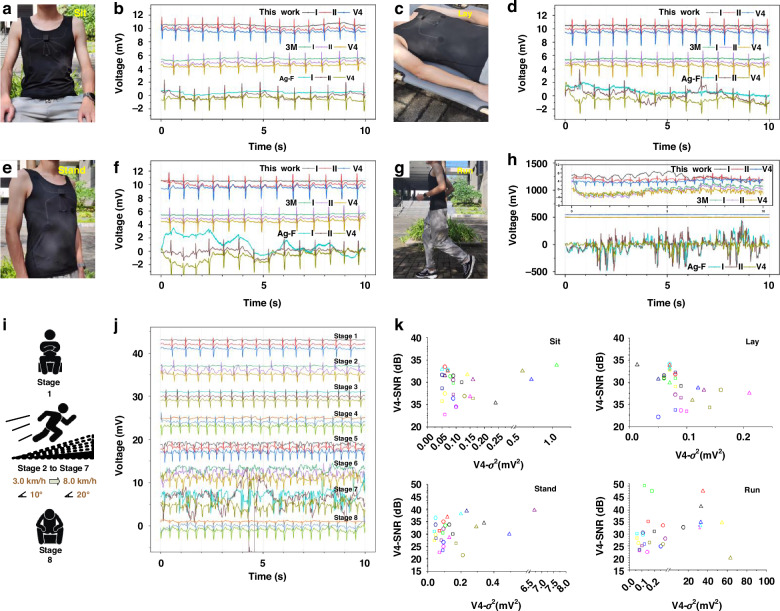
Daily dynamic/static ECG signal monitoring. The output signals of volunteers in **a**, **b** Seating state; **c**, **d** Laying state; **e**, **f** Stading state; **g**, **h** Running state and **(i**, **j)** Dynamic state. **k** The SNR and *σ*^2^ of ECG data calculated from 10 volunteers

The output signals of volunteers in seated state are displayed in Fig. 6a-b. The ECG signals measured by AgCF-S electrodes and 3 M electrodes are relatively good, and the characteristic peaks are clearly distinguished. However, the signal baseline oscillates significantly in pure AgCF electrodes, caused by the sliding between electrode-skin interface under respiration. Similar fluctuation occurs when the volunteers are in standing and lying state (Fig. 6c-f). Because the lying position is a relatively ideal state, the influence of muscle electrical signals and motion artifacts is small, and the recognition ability is improved in different electrodes. As shown in Fig. 6g-h, interference of signals is highly increased at lead I, because the volunteers’ muscles are tense while running. At this time, we can see that the AgCF-S electrodes have the most stable ECG curve and high-quality P, Q, R, S, and T waveforms. This stable output is resulted from the synergy in 3D multichannel structure and Kirigami structure at the electrode-skin interface. Specifically, i) keep a stable contact area due to self-adhesive silicone; ii) self-adaptive of Kirigami substrate to avoid the interfacial sliding because; and iii) discharge sweat from 3D multichannel to improve the interfacial contact. So, compared with pure AgCF and 3 M electrodes, AgCF-S electrodes can reduce motion artifacts and make P, Q, R, S, and T peaks easier to identify.

In the dynamic state such as sports training, sweat is produced during intense exercise to enhance heat dissipation. However, sweat is unfavorable to the conformal contact in electrode-skin interface, causing motion artifacts and even electrode desorption. Here, the volunteers jogged at a set pace (about 6 km/h). After 30 seconds, we collected ECG data of the 3-limb-lead ECG suit under exercise conditions. The volunteers followed the set speed and slope program for exercise, and the procedures are set as follows:

(1) Resting state;

(2) Class 1, speed 2.7 km /h, slope 10%, time 3 min;

(3) Level 2, speed 4.0 km /h, slope 12%, time 3 min;

(4) Level 3, speed 5.5 km /h, slope 14%, time 3 min;

(5) Level 4, speed 6.8 km /h, slope 18%, time 3 min;

(6) Level 5, speed 8.0 km/h, slope 18%, time 3 min;

(7) Level 6, speed 8.9 km /h, slope 20%, time 3 min;

(8) Recovery period, speed 2 km/h, slope 0%, time 3 min.

The ECG waveforms of different stages are shown in Fig. 6i. With the increase of exercise intensity, the collected ECG data is seriously disturbed. Volunteers in the stage 2-4 exercise, the 3-limb-lead ECG suit can obtain relatively good ECG waveform. In the stage 4, volunteers had begun to sweat, and the adhesion at the electrode-skin interface decreased. Entering stage 5, increased exercise intensity leads to increased sweating and more pronounced motion artifact interference in the ECG signal. As the speed of stage 6 increases, a clutter signal is introduced in the I and II leads due to force oscillations. In stage 7, the volunteers sweat violently, the electrode and skin slide each other, and the interface adhesion is almost invalid. Poor contact occurs and a larger clutter signal is introduced at the limb lead (Fig. 6j).

Statistical ECG data of 10 volunteers are extracted and compared, including the SNR and *σ*^2^, which are summarized in Fig. 6k and Tables S2-5. In some data, when the signal variance is close, the SNR of the AgCF-S electrode is close to or even greater than that of 3 M electrode, indicating that the signal-acquisition ability is close to that of wet electrode. The SNR and *σ*^2^ of the ECG signal collected by pure AgCF electrode is higher than that of other two electrodes, caused by the fluttering and moving artifacts of baseline. In addition to pure AgCF electrodes, both AgCF-S and 3 M electrodes can effectively identify QRS peak groups and calculate heart rate through filters. The jump variance of three electrodes is the same order of magnitude, but the values are changed in the 10 volunteers due to individual differences. In Table S6, we summarize the SNR of state-of-the-art ECG electrodes using active materials including CNT/PDMS^38^, CNT/graphene^31^, AgNWs/PU^23^, Zeolite/PDMS^14^, Ag-fiber/Silicone^39^, AgNPs/Ecoflex^40^, AgNWs/PDMS^41^, MWCNT/PDMS^42^, LIG/PDMS^43^ and APTES/PDMS^44^. In contrast, our ECG electrodes keep a relatively high SNR while being breathable and flexible.

**Fig. 7:**
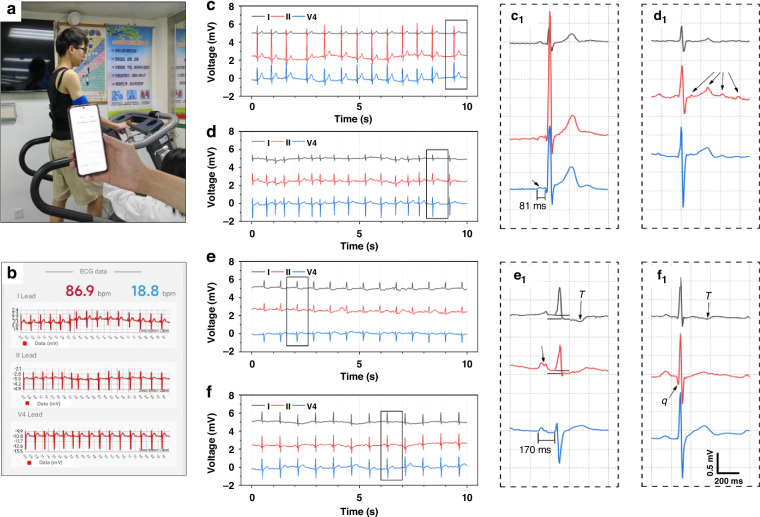
Basic ECG signal monitoring of typical heart diseases. **a**, **b** Patients were examined to verify the function of recognizing heart disease. ECG signals of **c** Volunteer No. 1 with arrhythmia, **d** Volunteer No. 2 with hypertension and diabetes, **e** Volunteer No. 3 had heart stent implantation and **(f)** Volunteer No. 4 with coronary heart disease

ECG examination is an important method to diagnose heart disease in clinic. One of the common cardiac problems in clinic is arrhythmia, which shows obvious abnormalities on the ECG data. A qualified ECG device equipped with a standard 3-limb lead can detect this issue. To evaluate the signal collection of our device and verify its function to discriminate cardiac diseases, we examined 4 hospitalized patients under the guidance of doctors (Fig. 7a). Volunteer No. 1 was diagnosed with arrhythmia, so the ECG P-R interval of ECG curve is short (Fig. 7c). The P-R interval is less than 120 ms, and the II lead amplitude is high. Volunteer No. 2 was diagnosed with hypertension and diabetes (Fig. 7d). The R-R interval was irregular in the ECG curve, and f wave appeared in the II and V4 leads, which could be judged as atrial fibrillation. Volunteer No. 3 had heart stent implantation experience, and the ECG waveform is shown in Fig. 7e. The S-T segment of the I and II leads was pressed down, and the T-wave of the I leads was inverted. Meanwhile, the P-wave widened in the II and V4 leads, and the P-R interval was close to 200 ms, because of the left atrial hypertrophy. Volunteer No. 2 was diagnosed with coronary heart disease. As shown in Fig. 7f, the overall rhythm of ECG waveform was normal. The enlarged image shows the T wave in the I lead was inverted, and the Q wave in the II lead was abnormal, indicating a previous experience of myocardial infarction (Fig. 7f). The specific symptoms of patients were accurately verified, demonstrating the huge potential in disease screening. From the above analysis, the ECG waveform detected by our ECG suit is consistent with the specific medical records. All the volunteers were all knowingly tested in a lying position using standard 3-limb-lead ECG suit, revealing the good compatibility between the electrode and skin. Thanks to the 3D multi-channel structure, the on-skin electrodes are air permeable, allowing for prolonged wear. The Kirigami structure of flexible substrate and self-adhesive silicone are beneficial to the accuracy of ECG signals.

## Conclusions

In this work, the AgCF-S dry adhesives are prepared by a self-template strategy consisting of a dissolving-curing-redissolving process. The 3D multi-channels act as water vapor path at the electrode-skin interface, allowing sweat to easily penetrated with an evaporation rate of 1.8 μg/mm^2^/min, and no sensitization occurs after 24 h of continuous adhesion. The AgCF-S electrodes are connected to the standard 3-limb-lead ECG suit to extract the signals under dynamic and static conditions such as sitting, standing, lying, and running. The I-III, aVR, aVL, aVF, V4 lead data are synchronously transmitted in real time through Bluetooth and displayed in the client. Due to the synergy of the 3D multi-channel structure of on-skin adhesive and Kirigami structure of flexible substrate, the AgCF-S electrode exhibits the most stable ECG curve and high-quality P, Q, R, S, T waveforms compared to the pure AgCF and 3 M electrodes. Some typical CVDs such as coronary heart disease, arrhythmia, myocardial infarction, etc., are detected by ECG equipment, to illustrate its huge potential in future medical electronics.

## Supplementary information

Supporting information

Video S1
