# Supplementary material for: Self-template manufacturing of on-skin electrodes with 3D multi-channel structure for standard 3-limb-lead ECG suit
Source: Microsyst Nanoeng. 2024 Dec 16;10:196. doi: 10.1038/s41378-024-00838-7 (PMC11649698; doi:10.1038/s41378-024-00838-7)
Supplement: Supplementary file 1 — Supporting information [file 41378_2024_838_MOESM1_ESM.pdf]

ASSOCIATED CONTENT

Supporting Information

Self-template manufacturing of on-skin electrodes with 3D multi-channel structure for standard 3-limb-lead ECG suit

Wentao Wang [a], *Longsheng Lu* [b], *Huan Ma* [c], *Zehong Li* [b], *Xiaoyu Lu* [b], *Yingxi Xie* [b,*]

[a] School of Automotive and Mechanical Engineering, Changsha University of Science and Technology, Changsha 410114, China

[b] Guangdong Key Laboratory of Precision Equipment and Manufacturing Technology, South China University of Technology, Guangzhou 510641, China

[c] Guangdong Cardiovascular Institute, Guangdong Provincial People's Hospital, Guangzhou 510080, China

[*] Corresponding authors: Xieyingxi@scut.edu.cn (Dr. Y. Xie)

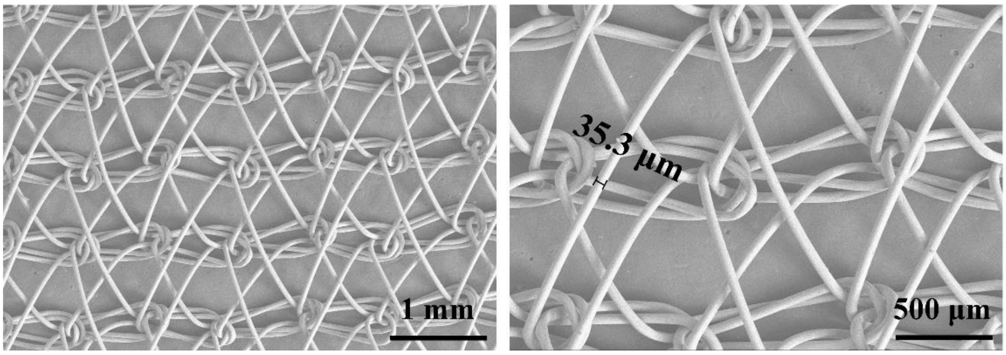

Fig. S1 Surface morphology of pure AgCFs.

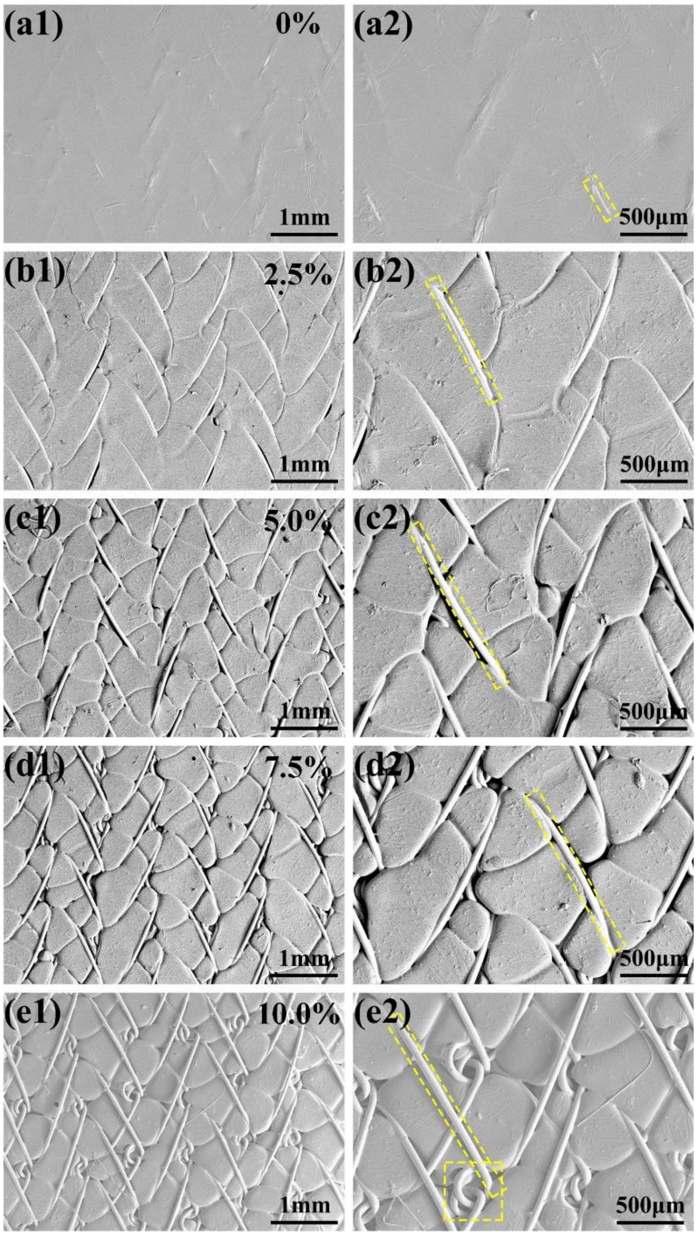

Fig. S2 Surface morphology of AgCF-S dry adhesive with different PVA concentration.

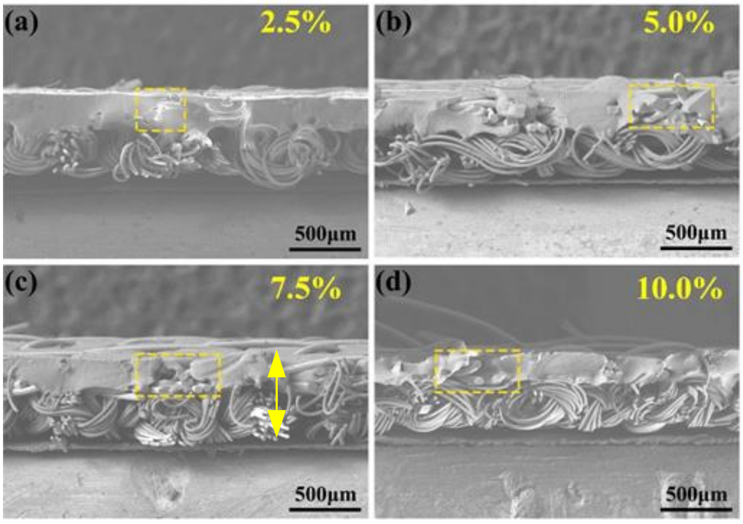

Fig. S3 Cross-section morphology of AgCF-S dry adhesive with different PVA concentration.

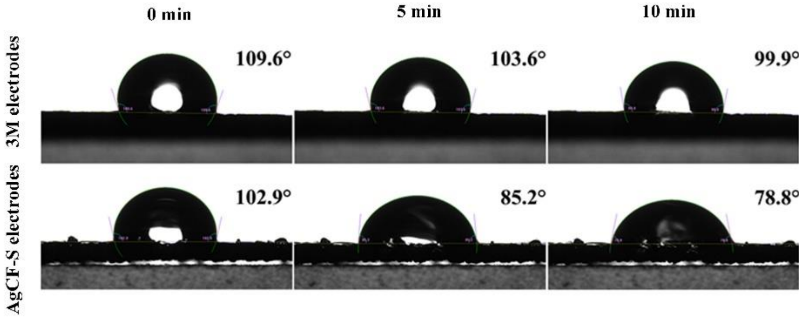

Fig. S4 Contact Angle of sweat on 3M electrode and AgCF/S dry adhesive.

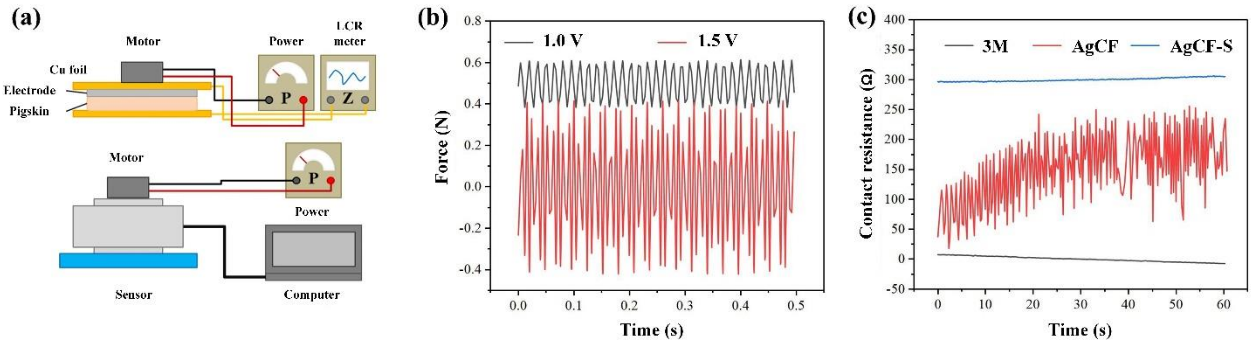

Fig. S5 Stability test of contact resistance. (a) Schematic diagram of experimental setup; (b) Forces under different voltages; (c) Contact resistance with pig skin at 30Hz.

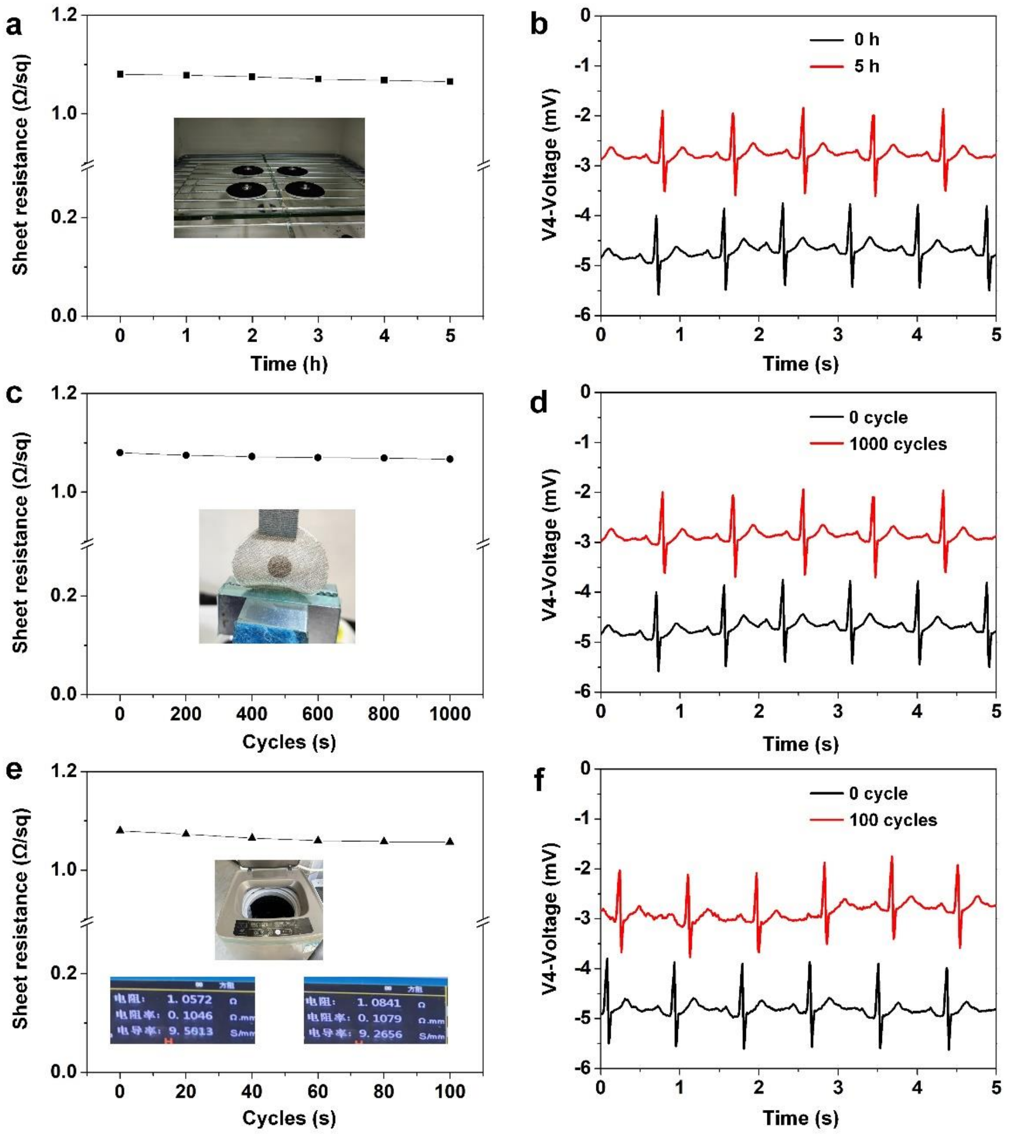

Fig. S6 The sheet resistance and ECG signals of the AgCF-S electrodes in (a) High-temperature of 80 ℃, (b) Repeated bending 1000 times and (c) Laundry of 100 times.

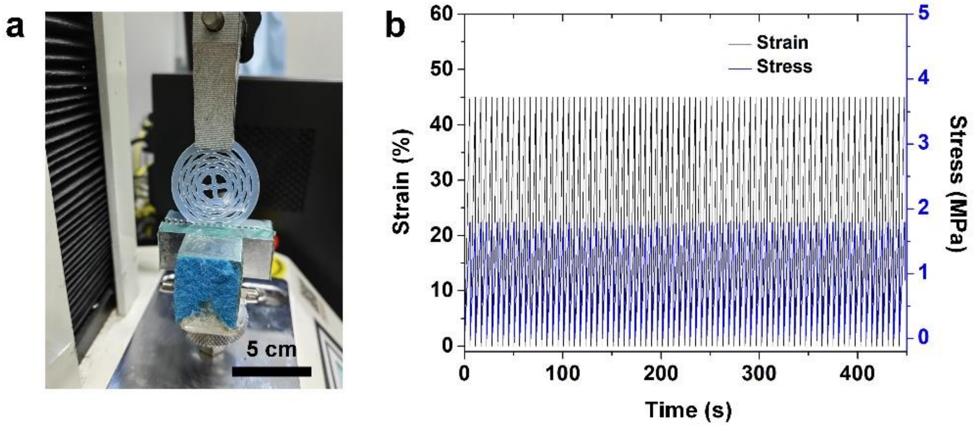

Fig. S7 The change of strain and stress during 100 times of stretch-recovery cycle at 0-50 %.

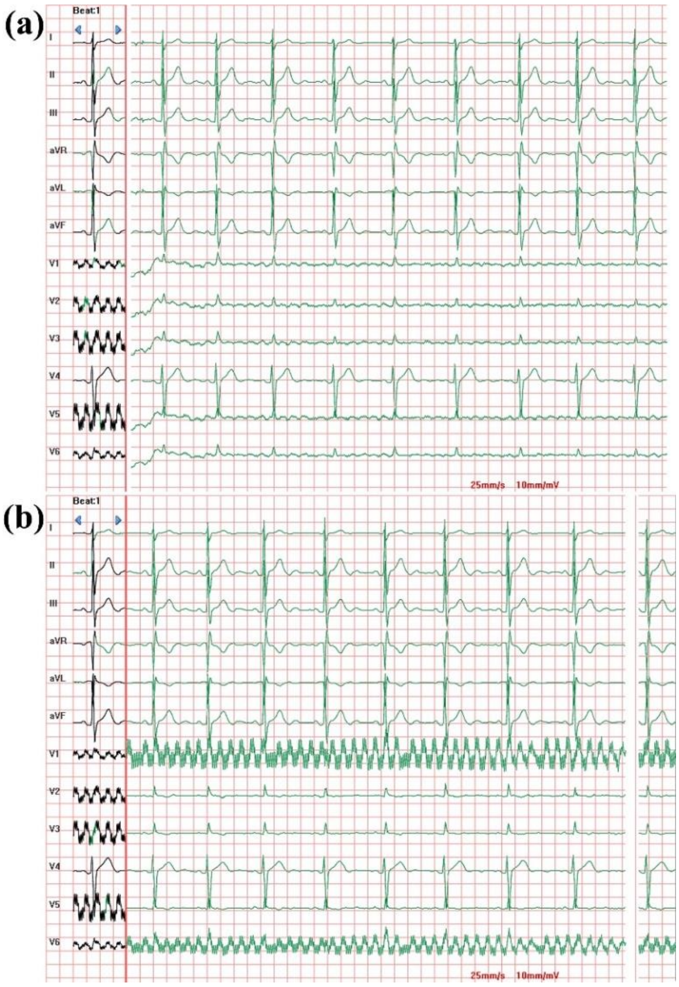

Fig. S8 (a)The 3M electrodes and (b) AgCF/S electrodes used in medical-grade ECG machine.

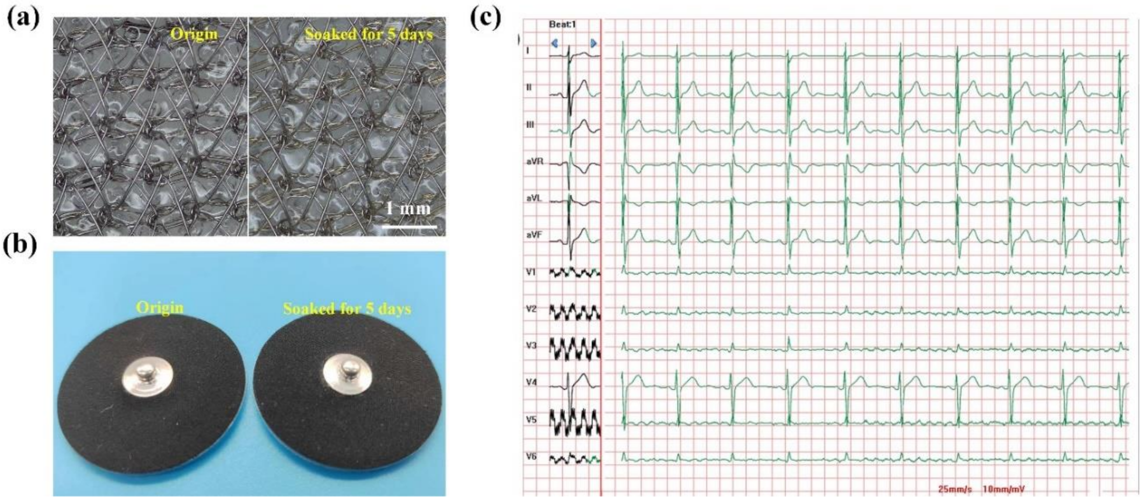

Fig. S9 The AgCF/S electrodes soaked in artificial sweat for 5 days. (a) Surface status. (b) electrode status. (c) ECG output

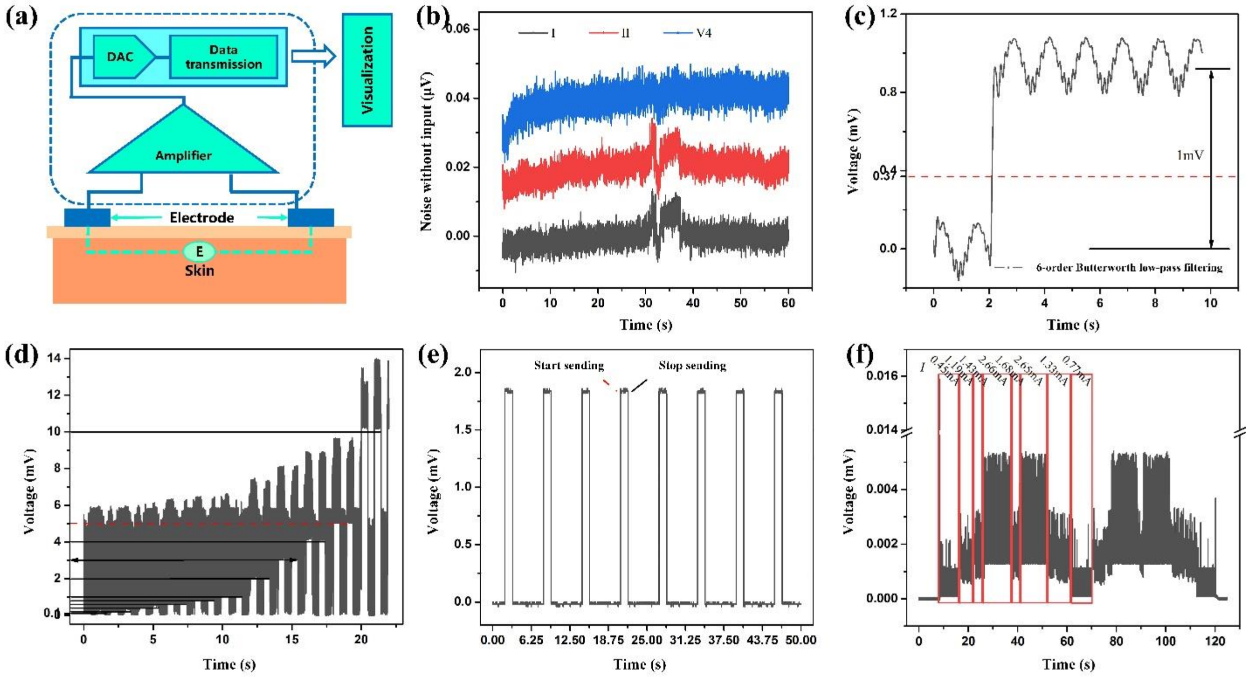

Fig. S10 Performance curve of ECG equipment. (a) Interfical contact model. (b) System noise and drift. (c) Differential input step response time constant. (d) Signal amplification linearity. (e) ECG acquisition timing accuracy. (f) Device current and effective values at each stage.

Table S1 Device performance statistics

Differential input impedance	Common mode rejection ratio	System noise/drift
500 MΩ	107 dB@50 Hz	<30 μV/<25 μV@60s
Dynamic input range	Bandwidth	Resolution
±400 mV	0-65 Hz	0.2 μV
Gain	Sampling rate	Time constant
3.5	160 sps	>5s
Working hours	Standby time	Low power wake-up time
~44h	6.5 days	<1s

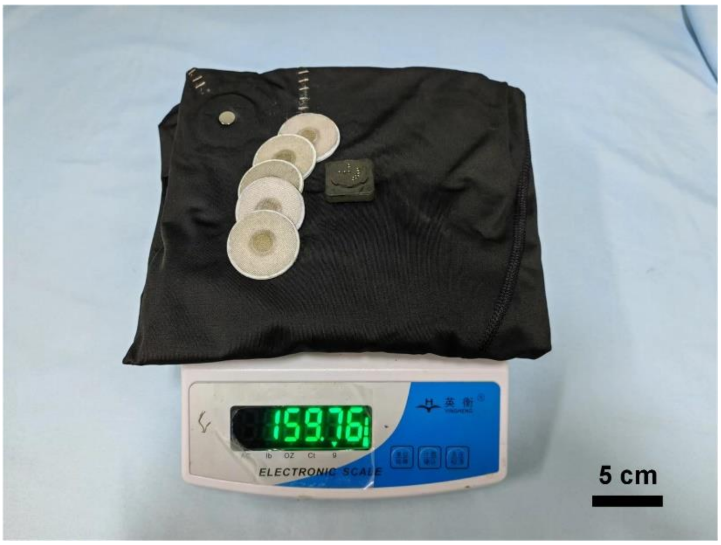

Fig. S11 The total mass of ECG suit and core machine is about 160 g

Method for calculating signal-to-noise ratio:

The signal noise part is defined as the signal component with a frequency above 50 Hz, and the useful signal part is defined as the signal component with a frequency of 0.5-50 Hz. The useful signal and noise signal are obtained by using IIR bandpass and high-pass filter provided by Origin, the filter order is 6, and the "forward-backward filter" is set. The SNR is given by the following expression:

$$SNR(dB) = 20log_{10}\frac{RMS_{useful}}{RMS_{noisy}} \qquad (1)$$

Where the RMS_{useful} is the root mean square of the useful signal part; RMS_{noisy} is the root mean square of the noise part. RMS is the square root of all signal sampling point values V_i, and the formula is as follows:

$$MS = \sqrt{\frac{\sum_{i=1}^{N} V_i^2}{N}} \qquad (2)$$

Where N is the total number of signal sampling points. Signal variance σ^2 describes the degree of variation in the signal waveform. For AC signals, a smaller σ^2 means a gentler signal, which is calculated as follows:

$$\sigma^2 = \frac{\sum_{i=1}^{N}(V_i - V)^2}{N} \qquad (3)$$

Where V is the average of all signal sampling point values V_i

Table S2 Statistical data of 10 volunteers in seating state

Code	V4-*SNR*(50 Hz)	V4-σ^2
1	29.19/29.34/32.47	0.09/0.06/0.13
2	33.16/30.14/32.39	0.07/0.08/0.07
3	30.92/29.20/28.19	0.08/0.06/0.07
4	23.77/21.59/27.10	0.08/0.05/0.12
5	33.03/32.75/31.68	0.07/0.07/0.07
6	23.45/22.70/25.98	0.10/0.09/0.21
7	29.07/29.74/31.16	0.08/0.06/0.07
8	24.33/26.66/24.59	0.14/0.16/0.11
9	32.25/29.83/29.03	0.08/0.06/0.05
10	26.52/25.69/26.62	0.09/0.08/0.13

Table S3 Statistical data of 10 volunteers in laying state

Code	V4-*SNR*(50 Hz)	V4-σ^2
1	30.02/29.75/25.36	0.12/0.09/0.24
2	31.52/31.90/32.53	0.06/0.06/0.07
3	29.86/29.60/33.82	0.09/0.08/1.06
4	24.42/24.93/30.60	0.10/0.09/0.72
5	32.58/31.20/32.88	0.07/0.07/0.05
6	22.76/23.41/26.78	0.06/0.10/0.15
7	25.74/25.91/31.70	0.05/0.06/0.14
8	26.37/25.40/32.53	0.16/0.13/0.60
9	28.66/29.93/31.41	0.05/0.05/0.06
10	27.26/28.79/30.56	0.09/0.09/0.16

Table S4 Statistical data of 10 volunteers in standing state

Code	V4-*SNR*(50 Hz)	V4-σ^2
1	30.01/28.68/29.24	0.15/0.11/0.29
2	31.32/29.89/31.76	0.07/0.08/0.10
3	30.46/28.29/46.34	0.09/0.09/7.06
4	23.48/22.43/25.06	0.09/0.08/0.42
5	31.05/31.49/33.18	0.04/0.04/0.17
6	22.63/21.15/23.90	0.07/0.08/0.11
7	27.45/24.90/36.54	0.04/0.04/0.72
8	26.25/18.75/27.84	0.17/0.18/0.25
9	28.32/28.64/34.53	0.05/0.04/0.20
10	27.47/27.05/34.97	0.08/0.08/0.55

Table S5 Statistical data of 10 volunteers in running state

Code	V4-*SNR*(50 Hz)	V4-σ^2
1	31.11/32.81/41.45	0.20/0.24/4.35
2	35.22/33.57/47.93	0.15/0.15/7.15
3	50.18/48.00/28.81	0.12/0.10/5656
4	25.77/24.84/34.85	0.12/0.14/4.00
5	30.16/29.77/33.33	0.06/0.06/3.36
6	23.11/22.46/32.67	0.08/0.08/1.08
7	28.32/26.23/34.72	0.06/0.04/34.87
8	26.45/25.73/19.94	0.16/0.15/47.32
9	23.41/30.57/29.44	0.08/0.06/310
10	25.09/28.07/40.21	0.10/0.16/1618

Table S6 Comparison of representative ECG electrodes with AgCF/S dry electrodes

Code	Materials	SNR
1	This work	28.5 dB vs 27.9 dB (Conv.)
2	CNT/aPDMS	4 mV vs 4mV (Conv.)[1]
3	CNT/graphene	0.2 mV vs 0 mV (Conv.)[2]
4	LIG/silicone	24.1 dB vs 21.6 dB (Conv.)[3]
5	AgNWs/PU	24.31 dB vs 24.86 dB (Conv.)[4]
6	Ag-fiber/Silicone	15.27 dB vs 15.34 dB (Conv.)[5]
7	AgNPs/ecoflex	10.57 dB vs11.05 dB (Conv.)[6]
8	AgNWs/aPDMS	500 μV vs 450 μV (Conv.)[7]
9	Zeolite-PDMS	42.9 dB vs NA dB (Conv.)[8]
10	MWCNT/PDMS	35.8-38.7 dB vs NA dB (Conv.)[9]
11	APTES/PDMS	21.82 vs 21.82 dB (Conv.)[10]

References

1. Lee, S. M. *et al.* Self-adhesive epidermal carbon nanotube electronics for tether-free long-term continuous recording of biosignals. *Sci. Rep.* **4**, 6074 (2014).
2. Kim, T., Park, J., Sohn, J., Cho, D. & Jeon, S. Bioinspired, Highly Stretchable, and Conductive Dry Adhesives Based on 1D-2D Hybrid Carbon Nanocomposites for All-in-One ECG Electrodes. *ACS Nano* **10**, 4770–4778 (2016).
3. Sun, B. *et al.* Gas-Permeable, Multifunctional On-Skin Electronics Based on Laser-Induced Porous Graphene and Sugar-Templated Elastomer Sponges. *Adv. Mater.* **30**, 1804327 (2018).
4. Jiang, Z. *et al.* Highly Stretchable Metallic Nanowire Networks Reinforced by the Underlying Randomly Distributed Elastic Polymer Nanofibers via Interfacial Adhesion Improvement. *Adv. Mater.* **31**, 1–9 (2019).
5. Xie, Y., Lu, L., Wang, W. & Ma, H. Wearable multilead ECG sensing systems using on-skin stretchable and breathable dry adhesives. *Bio-Design Manuf.* **7**, 167–180 (2024).
6. Guo, W. *et al.* Matrix-Independent Highly Conductive Composites for Electrodes and Interconnects in Stretchable Electronics. *ACS Appl. Mater. Interfaces* **11**, 8567–8575 (2019).
7. Kim, J. H., Kim, S. R., Kil, H. J., Kim, Y. C. & Park, J. W. Highly Conformable, Transparent Electrodes for Epidermal Electronics. *Nano Lett.* **18**, 4531–4540 (2018).

8. Pullano, S. A., Kota, V. D., Kakaraparty, K., Fiorillo, A. S. & Mahbub, I. Optically Unobtrusive Zeolite-Based Dry Electrodes for Wearable ECG Monitoring. *IEEE Sens. J.* **22**, 10630–10639 (2022).
9. Tasneem, N. T., Pullano, S. A., Critello, C. D., Fiorillo, A. S. & Mahbub, I. A low-power on-chip ECG monitoring system based on MWCNT/PDMS dry electrodes. *IEEE Sens. J.* **20**, 12799–12806 (2020).
10. Ying Meng, Zhenbo Li, J. C. A flexible dry electrode based on APTES-anchored PDMS. *Microsyst Technol* **22**, 2027–2034 (2016).
